# Pharmacophore Models and Pharmacophore-Based Virtual Screening: Concepts and Applications Exemplified on Hydroxysteroid Dehydrogenases

**DOI:** 10.3390/molecules201219880

**Published:** 2015-12-19

**Authors:** Teresa Kaserer, Katharina R. Beck, Muhammad Akram, Alex Odermatt, Daniela Schuster

**Affiliations:** 1 Institute of Pharmacy/Pharmaceutical Chemistry and Center for Molecular Biosciences Innsbruck (CMBI), Computer Aided Molecular Design Group, University of Innsbruck, Innrain 80/82, 6020 Innsbruck, Austria; Teresa.Kaserer@uibk.ac.at (T.K.); Muhammad.Akram@uibk.ac.at (M.A.); 2 Swiss Center for Applied Human Toxicology and Division of Molecular and Systems Toxicology, Department of Pharmaceutical Sciences, Pharmacenter, University of Basel, Klingelbergstrasse 50, 4056 Basel, Switzerland; Katharina.Beck@unibas.ch

**Keywords:** pharmacophore, virtual screening, ligand protein interactions, hydroxysteroid dehydrogenase, oxidoreductase

## Abstract

Computational methods are well-established tools in the drug discovery process and can be employed for a variety of tasks. Common applications include lead identification and scaffold hopping, as well as lead optimization by structure-activity relationship analysis and selectivity profiling. In addition, compound-target interactions associated with potentially harmful effects can be identified and investigated. This review focuses on pharmacophore-based virtual screening campaigns specifically addressing the target class of hydroxysteroid dehydrogenases. Many members of this enzyme family are associated with specific pathological conditions, and pharmacological modulation of their activity may represent promising therapeutic strategies. On the other hand, unintended interference with their biological functions, e.g., upon inhibition by xenobiotics, can disrupt steroid hormone-mediated effects, thereby contributing to the development and progression of major diseases. Besides a general introduction to pharmacophore modeling and pharmacophore-based virtual screening, exemplary case studies from the field of short-chain dehydrogenase/reductase (SDR) research are presented. These success stories highlight the suitability of pharmacophore modeling for the various application fields and suggest its application also in futures studies.

## 1. Introduction

### Pharmacophore Modeling

**Figure 1:**
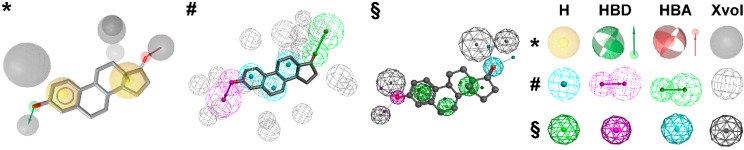
Pharmacophore models based on the estrogen equilin co-crystallized with 17β-hydroxysteroid dehydrogenase type 1 (PDB entry 1EQU [5]) and generated with LigandScout [6] (*), Discovery Studio [7] (#), and Molecular Operating Environment (MOE) [8] (*§*). H, hydrophobic feature; HBD, hydrogen bond donor; HBA, hydrogen bond acceptor; XVols, exclusion volume.

The concept of “pharmacophores” dates back to the late 19th century, when Paul Ehrlich suggested that specific groups within a molecule are responsible for its biological activity [1,2]. The pharmacophore definition, as currently used, was developed over time, with many researchers actively participating in the process (for a detailed history of pharmacophores, please refer to Güner and Bowen [2]). However, Schueler provided the basis for our modern understanding of a pharmacophore [2,3], which is defined by the International Union of Pure and Applied Chemistry (IUPAC) as “the ensemble of steric and electronic features that is necessary to ensure the optimal supra-molecular interactions with a specific biological target structure and to trigger (or to block) its biological response” [4]. According to this definition, the interaction patterns of bioactive molecules with their targets are represented via a three-dimensional (3D) arrangement of abstract features that define interaction types rather than specific functional groups. These interaction types can, for example, include the formation of hydrogen bonds, charged interactions, metal interactions, or hydrophobic (H) and aromatic (AR) contacts (Figure 1). Besides that, many pharmacophore modeling programs allow for the addition of steric constraints. These so-called exclusion volumes (XVols) mimic the geometry of the binding pocket and prevent the mapping of compounds that would be inactive in the experimental assessment due to clashes with the protein surface. In its entirety, a pharmacophore model represents one binding mode of ligands with a specific target, as exemplified on 17β-hydroxysteroid dehydrogenase (HSD) type 1 (Figure 1).

**Figure 2:**
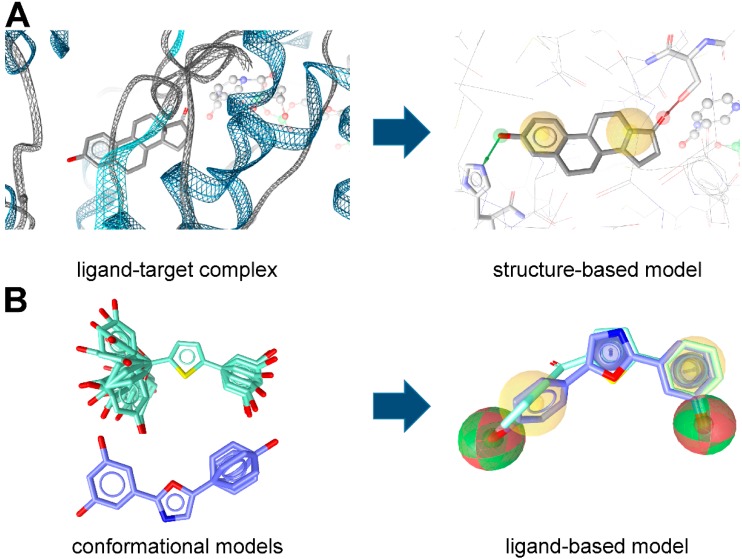
(**A**) Structure- and (**B**) ligand-based pharmacophore model generation with LigandScout. (**A**) Based on the complex of equilin bound to 17β-HSD1 (PDB entry 1EQU [5]), an initial pharmacophore model is created automatically; (**B**) Conformational models of known 17β-HSD1 ligands [13,14] are used to align the compounds and extract pharmacophore features they share.

Pharmacophore models can be generated using two different approaches (Figure 2) depending on the input data employed for model construction. In the structure-based approach, the interaction pattern of a molecule and its targets are directly extracted from experimentally determined ligand-target complexes (Figure 2A). An important source for these complexes, e.g., derived from NMR-spectroscopy or X-ray crystallography, represents the Protein Data Bank (PDB, www.pdb.org) [9]. To date (access date 2 November 2015), more than 113,000 macromolecular structures are stored in this online repository. However, not all of these structures were solved in a complex with a bound ligand, and in the case of induced fit, the binding of different ligands to an enzyme or receptor can lead to different interactions that are not covered by a single structure. To address this limitation, some pharmacophore modeling programs, e.g., Discovery Studio [7] and LigandScout [6], also provide tools to create pharmacophore models based exclusively on the topology of the binding site and in the absence of a ligand [10]. In Discovery Studio, for example, the binding site can be defined manually by selecting residues within the desired cavity or by applying implemented binding site identification tools. Once the binding site is defined, the program automatically calculates pharmacophore features based on the residues lining the active site. This initial ensemble of pharmacophore features can then be adapted to construct the final hypothesis [10]. In addition, structure-based pharmacophore models can also be generated with computationally derived ligand-target complexes. In the course of a docking run, known active compounds are fitted into the empty binding pocket of the target [11]. These docked binding poses can then directly be employed to extract the interaction patterns. For further refinement of the initial docking poses, molecular dynamics (MD) simulations can be conducted [12] prior to model generation.

In the course of ligand-based modeling, three-dimensional (3D) structures of two or more known active molecules are aligned and common pharmacophore features shared among these training set molecules are identified (Figure 2B). In a ligand-based approach, all of the common chemical features from the pharmacophore have to be presumed as essential, whereas in a structure-based approach, it can be considered whether a chemical feature of a molecule is directly involved in the ligand binding or not.

**Figure 3:**
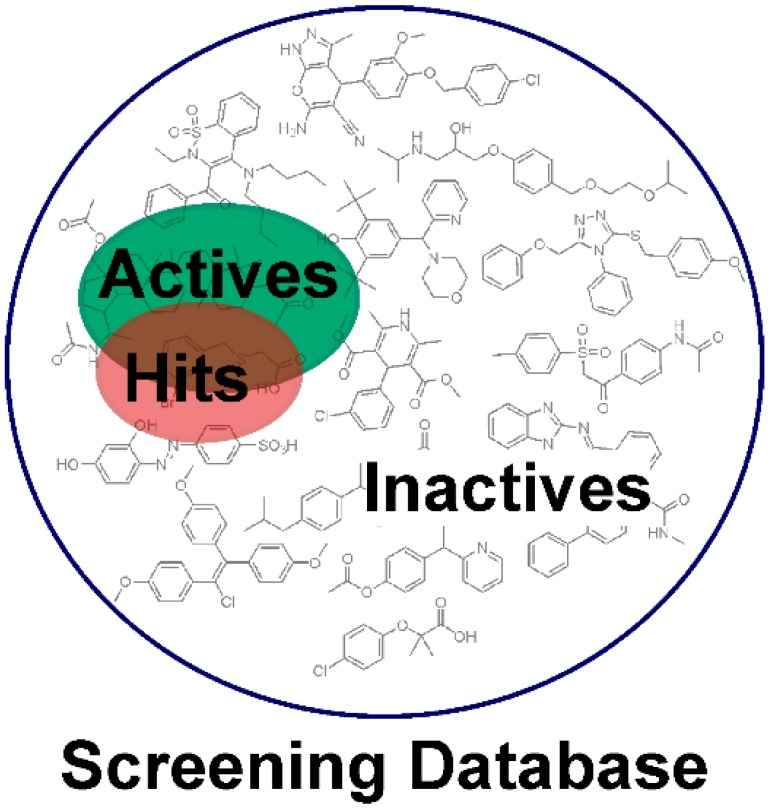
Enrichment of active molecules in the virtual hit list. Usually, the majority of compounds in a screening database are inactive molecules, while a small pool of bioactive molecules is contained. Pharmacophore-based virtual screening can help to enrich active molecules in the hit list compared to a random selection of test compounds.

Usually, datasets containing known active and inactive molecules are employed to assess the quality of the developed models. These datasets need to be designed carefully, because they largely influence the quality of the model and, accordingly, the success of the study. Only active molecules should be included, for which the direct interaction has been experimentally proven [15,16], e.g., by receptor binding or enzyme activity assays on isolated or recombinant proteins. Cell-based assays should be avoided in this context, because many factors other than interaction with the target can influence the results: Active compounds may potentially exert their effect via other mechanisms than the intended one, whereas on the other hand, inactive compounds may actually interact with the target, but due to poor pharmacokinetic properties, this cannot be detected. In addition, appropriate activity cut-offs need to be defined to avoid the inclusion of compounds with a low binding affinity and high EC_50_/IC_50_ values (which may even be classified as “inactive”). Finally, the dataset should contain structurally diverse molecules [17] whenever possible. Preferably, experimentally confirmed inactive compounds should be included in the “inactives” dataset used for the theoretical validation [17,18]. Besides the original literature, several public compound repositories such as ChEMBL [19], Drugbank [20], or OpenPHACTS [21] can be explored for target-based activity data of compounds. In addition, several high-throughput screening (HTS) initiatives such as ToxCast [22], Tox21 [23], and PubChem Bioassay [24] provide a valuable resource for both active and inactive molecules. Whenever no or only a limited number of known inactive molecules are available, so-called decoys (compounds with unknown biological activity but assumed to be inactive) might be employed. These decoy-datasets need to be adapted for every target and should contain compounds with similar one-dimensional (1D) properties [25,26,27] but different topologies compared to the known active molecules. These properties can include the number of hydrogen bond donors (HBDs), the number of hydrogen bond acceptors (HBAs), the number of non-polar atoms [25], molecular weight, logP, and the number of rotatable bonds [27]. The Directory of Useful Decoys, Enhanced (DUD-E) [28] provides a free service (http://dude.docking.org), where optimized decoys are generated based on the smiles codes of the uploaded active molecules. In general, a ratio of about 1:50 for the number of active molecules and decoys is recommended [28]. This should reflect the prospective screening database, where usually only a few active molecules are also distributed among a vast amount of inactive molecules (Figure 3).

The preliminary models generated with both approaches need further improvement in the majority of cases [16,29] to facilitate the recovery of the active molecules and concomitantly exclude the inactive compounds in the dataset from the hit list. Basic model refinement steps include the deletion or addition of pharmacophore features and adaptations concerning the feature weight and size. Selected features can also be defined as optional and, therefore, can but do not have to be mapped by a molecule. In addition, a user-defined number of omitted features can be specified in many pharmacophore modeling programs. More sophisticated modifications comprise the modification of feature definitions, *i.e.*, the functional groups covered by a pharmacophore feature.

**Figure 4:**
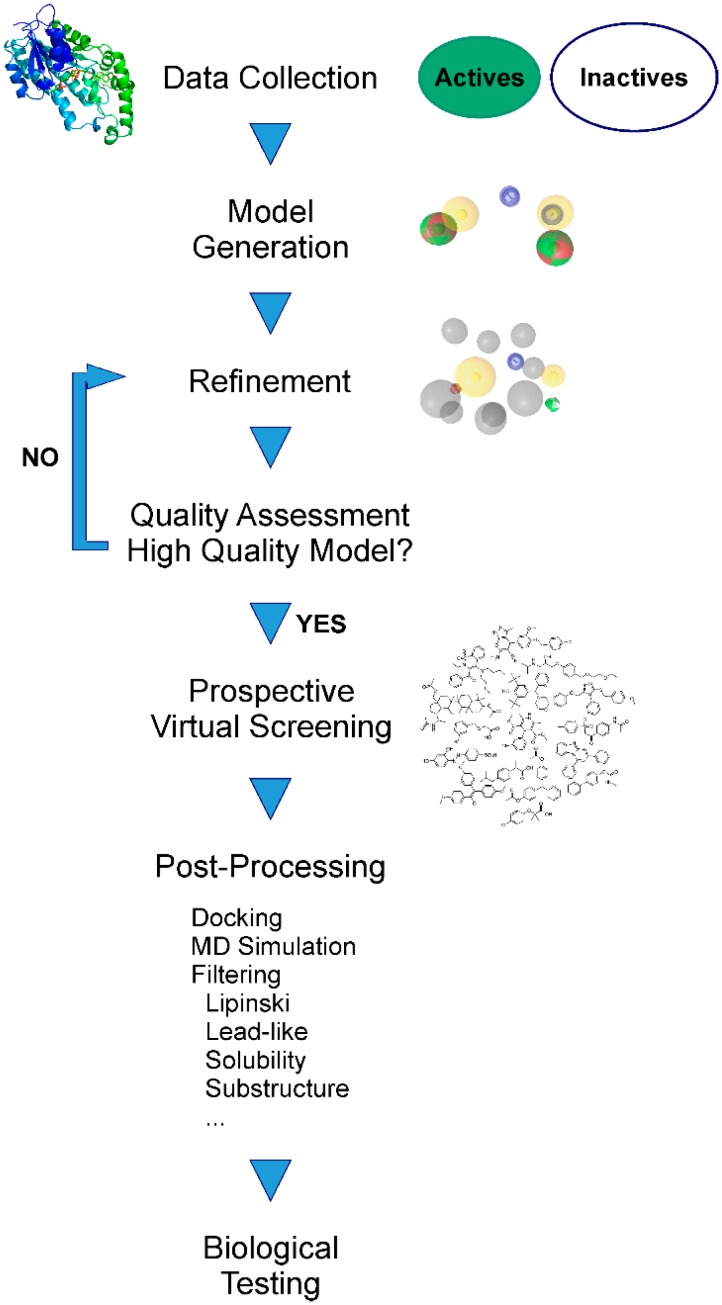
The different consecutive steps in pharmacophore model generation, refinement, and prospective application.

The aim of pharmacophore-based virtual screening (VS) is to enrich active molecules in a screening database in the virtual hit list (Figure 3). Multiple quality metrics are available that help to evaluate the quality of the developed pharmacophore model, for example the enrichment factor [30] (the enrichment of active molecules compared to random selection), yield of actives (the percentage of active compounds in the virtual hit list), specificity (the ability to exclude inactive compounds) and sensitivity (the ability to identify active molecules), and the area under the curve of the Receiver Operating Characteristic plot (ROC-AUC) [31]. For detailed descriptions of commonly applied quality parameters we refer to earlier work [15,16,26,32]. The ultimate proof of a model’s quality and value, *i.e.*, whether it is indeed capable of proposing novel active molecules, can, however, only be determined in a prospective experiment, as will be explained in more detail below. A workflow summarizing the individual steps of pharmacophore model generation and application is depicted in Figure 4.

As outlined below, refined, high quality pharmacophore models can then be employed for multiple tasks.

## 2. Applications of Pharmacophore-Based VS

In the course of a VS run, a pharmacophore model is screened against large chemical libraries, and molecules mapping the model are collected in a virtual hit list. These molecules fulfill the requirements of the model and therefore have a high likelihood to be active in the experimental testing. Accordingly, VS can be used to filter promising compounds out of large compound collections and enrich active molecules in chemical databases selected for experimental investigations. VS is considered a valuable support for classical HTS campaigns [33,34], because true positive hit rates are usually much higher than in those “random” testing strategies [35,36,37]. Reported hit rates from prospective pharmacophore-based virtual screening vary between individual studies, but are typically in the range of 5% to 40% (an excellent collection of prospective studies has been presented earlier [16]). On the other side, the hit rates of identifying active molecules upon random selection of test compounds are typically below 1% and have been described, for example, as 0.55% for glycogen synthase kinase-3β [36], 0.075% for peroxisome proliferator-activated receptor (PPAR) γ [38], and 0.021% for protein tyrosine phosphatase-1B [37].

### 2.1. Drug Discovery

Pharmacophore-based VS is widely applied in different steps of the drug discovery process and facilitates the initial selection of compound classes as well as the optimization of compound properties as outlined below.

#### 2.1.1. Lead Identification

The most common application of pharmacophore-based virtual screening concerns lead identification, the so-called cherry-picking approach. Virtual screening is often deployed in these projects to prioritize molecules for testing and minimizing the number of compounds to be investigated in biological screens. The ultimate aim is the identification of novel lead compounds for a specific disease-related target, which can be developed into drug candidates for the treatment of the intended disease, with numerous studies during the last years describing such applications [39,40,41,42,43,44]. For example, Ha *et al.* reported the discovery of novel ligands for the chemokine receptor CXCR2 by using a ligand-based pharmacophore modeling approach [45]. In the course of a pharmacophore-based virtual screening for novel histamine H_3_ receptor antagonists, Lepailleur *et al.* identified novel compounds additionally binding to the 5HT_4_ receptor [46]. Both activities were considered beneficial for the treatment of Alzheimer’s disease and the authors were the first to report compounds with this dual mechanism of action [46].

#### 2.1.2. Structure-Activity Relationships

As mentioned in the introduction, a pharmacophore model represents the putative binding mode of active molecules to their target. It therefore describes the crucial functionalities required for a compound’s activity. A pharmacophore model is trained to discriminate between active and inactive molecules (in the best case even between members of the same chemical series), which makes it highly valuable for establishing structure-activity relationships (SARs). Differences in the experimentally observed biological activities of a set of compounds can be rationalized based on the presence/absence of chemical groups, represented by pharmacophore features, in the respective molecules. SARs can be established during model building, thereby elucidating the underlying mechanisms for the (absent) biological activity. For example, Ferreira *et al.* employed pharmacophore models to elucidate important features responsible for the interaction of compounds with the P-glycoprotein drug binding site [47]. Previous studies suggested a crucial role for a nitrogen atom in the modulators; however, active constituents from *Euphorbia* species isolated in-house did not contain such a moiety. The authors generated multiple refined pharmacophore models and evaluated them against a dataset of literature-derived modulators, the in-house collection, and inactive molecules. Their final model highlighted the important role of hydrophobic contacts and the presence of a HBA feature for P-glycoprotein modulators and showed that mapping of the most active compounds was also preserved when a further HBA/HBD feature was added [47]. In addition, pharmacophore models can be employed to reflect previously elucidated SARs for the identification of novel bioactive molecules. In 2002, Flohr *et al.* used the endogenous peptide urotensin II and synthetic analogues to experimentally identify interactions that are crucial for binding to the urotensin II receptor [48]. Based on the established SAR, pharmacophore models were built and employed to screen a chemical library containing small drug-like compounds. Subsequent experimental testing of the virtual hits led to the identification of six novel scaffold classes, which, importantly, contained non-peptic molecules [48].

#### 2.1.3. Scaffold Hopping

A pharmacophore feature describes abstract chemical functionalities rather than specific functional groups. Additionally, pharmacophore models only demand local functional similarity of active compounds and virtual hits at 3D locations essential for biological activity. Therefore, there are no specifications concerning the actual two-dimensional (2D) structures of mapping compounds. Although the composition of a pharmacophore model is influenced by the 2D structure of the molecules employed for model generation and refinement, it still allows for mapping of structurally distinct hits. This makes pharmacophore modeling broadly applicable for the investigation of molecules originating from a diverse chemical space such as natural products and synthetic compounds. Importantly, it also allows for the identification of novel scaffolds that have not been associated with the target of interest before, a strategy that is called scaffold hopping. An earlier review extensively discussed pharmacophore modeling in the context of scaffold hopping [49]. A recent study employed pharmacophore modeling for the discovery of novel transient receptor potential vanilloid type 1 channel ligands [50]. Although the initial hits only weakly interacted with the target, they represent an interesting starting point for further chemical optimization. Such studies mostly emphasized novel chemical scaffolds and retrieved low similarity scores compared to the highly active compounds in the theoretical validation dataset [50].

Scaffold hopping is certainly relevant for the pharmaceutical industry that needs to explore compounds which are not yet covered by intellectual property issues. Of relevance for the general public, scaffold hopping facilitates the identification of chemicals with only limited available data. This is often the case for environmental pollutants and chemicals from consumer products that are often not drug-like by their nature.

#### 2.1.4. Selectivity Profiling

For some projects, it may be of the utmost importance to identify compounds that selectively modulate the activity of one or more isoforms of an enzyme (family) to trigger the desired biological effect. For example, steroidal core structures are frequently found in endogenous and exogenous bioactive compounds; however, these compounds often lack selectivity. To identify selective compounds, specific chemical substitutions leading to additional hydrophobic or ionic interactions and hydrogen bonds have to be implemented. It has to be emphasized that these specific chemical modifications allow for distinguishing between the enzyme of interest and its related enzymes.

For example, 17β-HSD1 inhibitors are promising drug candidates for the treatment of hormone-sensitive breast cancer as well as endometriosis because they block the activation of estrone to the highly potent endogenous estrogen receptor (ER) agonist estradiol [51,52,53]. On the other side, the converse reaction, (*i.e.*, inactivation of estradiol) mediated via 17β-HSD2, should not be blocked by these molecules. Ideally, bioassays of all relevant members within a given protein family would be employed to assess a compound’s selectivity. Additionally, proteins sharing structural similarity in the domain that contains the ligand binding pocket rather than sequence similarity should be considered in the selectivity assessment of compounds [54,55]. Thus, a huge number of proteins need to be covered in this resource- and time-consuming approach. In a first step, parallel screening using a large collection of pharmacophores, covering the most relevant proteins, allows for an initial characterization of a compound’s activity profile and facilitates the prioritization of the bioassays to be chosen for further biological analyses.

However, selectivity may not be limited to different isoforms. As exemplified by a study from Guasch *et al.*, it can even address the biological effect exerted via the same target [56]. The authors focused on the exclusive discovery of novel PPARγ partial agonists. The retrieval of full agonists was avoided to prevent the side effects accompanying full receptor activation. For this purpose, a pharmacophore model for full agonists (called the anti-pharmacophore) was generated and used to remove all potential full agonists from the screening database. In the second step, a partial agonist pharmacophore model was applied to identify potential partial agonists in the compound library. After several additional filtering steps, eight compounds were finally subjected to biological testing and five of them could be confirmed as novel PPARγ ligands displaying partial agonistic effects [56].

#### 2.1.5. Combination with Other Techniques

Pharmacophore models are also often used together with other methods to further increase the number of active molecules in the hit list *via* the application of a consensus approach. Commonly employed combinations comprise docking, shape-based modeling, and MD simulation.

In addition, a number of filters are available that help to limit the virtual hits to those with the desired properties and eliminate unwanted actions or molecules. Probably the most prominent filter represents the Lipinski’s, describing properties that are shared by approved and orally administered drugs [57]. In particular, these comprise a number of ≤5 HBDs, ≤10 HBAs, a molecular weight of ≤500, and a cLogP ≤5. Since all descriptors are either five or a multiple of five, Lipinski *et al.* referred to it as the “rule of five”. Although the rule of five was initially developed to predict the oral bioavailability of molecules, it is also widely applied as a general drug-like filter. Veber *et al.* suggested two other criteria for the oral bioavailability of compounds: First, compounds should have a number of ≤10 rotatable bonds and, second, either a polar surface area of ≤140 Å^2^ or ≤12 HBAs and HBDs [58].

In analogy to Lipinski’s rule of five, Congreve *et al.* introduced the “rule of three” for the identification of promising hit compounds in fragment-based drug discovery [59]. Their analysis revealed that most of the small compounds that were successfully optimized to potent lead-like candidates had a molecular weight of ≤300, a number of HBDs ≤3, a number of HBAs ≤3, and a cLogP ≤3 [59].

More recently, a substructure filter was developed to identify highly problematic compounds that notoriously produce false positive assay read-outs [60]. Baell and Holloway analyzed high-throughput testing results and observed that a group of molecules were prone to unspecifically interfere with some experimental test systems. The subsequently developed substructure filter can help to detect these pan-assay-interference compounds (PAINS) [60] prior to spending time and resources in investigating and optimizing such molecules [61].

Multiple of these methods and filters can be included as well. As an example, Noha *et al.* employed a variety of computational techniques in a sequential manner to identify novel inhibitors of microsomal prostaglandin E_2_ synthase-1 [62]. The workflow included multiple prefilters, among them also the Lipinski filter, a pharmacophore-based virtual screening procedure, and molecular docking. Out of the 17 molecules finally selected for testing, two showed good activity in the experimental assay, and two further had moderate effects. Temml *et al.* used a combination of pharmacophore- and shape-based virtual screening to identify novel liver X receptor agonists [44]. In their study mentioned above [56], Guasch *et al.* not only applied pharmacophore models, but also a multistep protocol comprised of electrostatic and shape similarity and molecular docking to identify novel PPARγ partial agonists.

### 2.2. The Short-Chain Dehydrogenase/Reductase Superfamily

**Figure 5:**
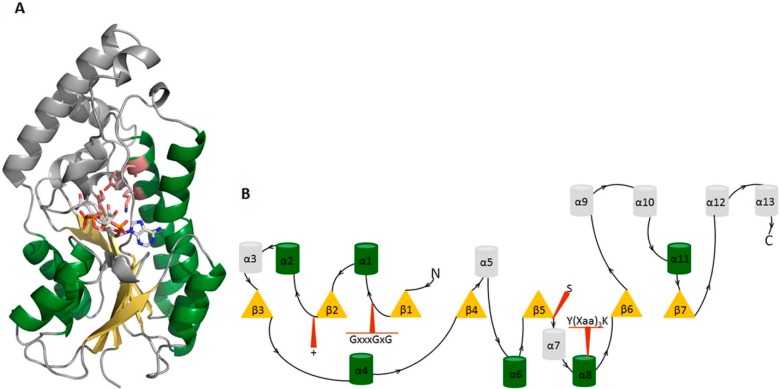
The general structure of SDR enzymes exemplified on 17β-HSD1 (PDB entry 1EQU [5]). (**A**) The Rossmann fold consists of parallel stranded β-sheets (yellow), which are flanked by α-helices on both sides (green). This structural domain forms the binding site of the co-factor NADP+. The residues Tyr155 and Lys159 of the Tyr-(Xaa)3-Lys motif as well as the conserved Ser142 are highlighted in rose; (**B**) 2D depiction of 17β-HSD1 (PDB entry 1EQU). Yellow triangles display β-sheets and barrel symbols α-helices. Apart from the Rossmann fold, structurally conserved regions are highlighted in red. The conserved glycine-rich motif GxxxGxG is important for cofactor binding and the + indicates a positive charged residue crucial for cofactor (NADP+) stabilization.

The short-chain dehydrogenase/reductase (SDR) enzyme family are nicotinamide adenine dinucleotide NAD (phosphate (P))-dependent enzymes sharing a common core structure of up to seven parallel stranded β–sheets flanked by three to four α–helices on each side, the so-called Rossmann fold, for NAD(P) binding and a catalytic center characterized by a Tyr-(Xaa)_3_-Lys motif. This motif is often found in combination with a conserved serine residue that stabilizes the orientation of the bound substrate (Figure 5) [63]. SDRs typically share a low sequence identity between 20%–30%, but with considerable structural similarity in the core domain.

The SDR family contains HSDs that play key roles in adrenal and gonadal steroidogenesis as well as in the metabolism of steroids in peripheral tissues [64]. Some of these HSDs are considered as promising therapeutic targets for the treatment of estrogen- and androgen-dependent diseases such as osteoporosis, endometriosis, and breast and prostate cancer, and other enzymes gained interest regarding the treatment of corticosteroid-related diseases such as diabetes, visceral obesity and dyslipidemia, atherosclerosis, wound healing, glaucoma, neurodegenerative disease, and cognitive impairment [53,65,66,67].

The development of specific SDR inhibitors needs to take into account the structural similarity of the various SDR enzymes in order to exclude the inhibition of members causing adverse effects, so-called off-targets. Suitable enzyme activity assays are fundamental for selectivity testing of potential inhibitors. Koch *et al.* proposed that structural similarity rather than primary sequence similarity should be chosen as the criterion for whether a certain chemical affects the activity of a related enzyme [54]. Therefore, the closest structurally related enzymes should be included for selectivity testing—using pharmacophore models and cell-based assays. Another application of the modeling approaches is the identification of toxic xenobiotics including industrial and environmentally relevant chemicals [68,69,70]. The role of several SDRs in xenobiotics metabolism and in steroid synthesis and metabolism makes them prone as targets for endocrine disruption [71,72,73,74,75,76].

## 3. Examples from the SDR Family

### 3.1. 11β-Hydroxysteroid Dehydrogenase Type 1

**Figure 6:**
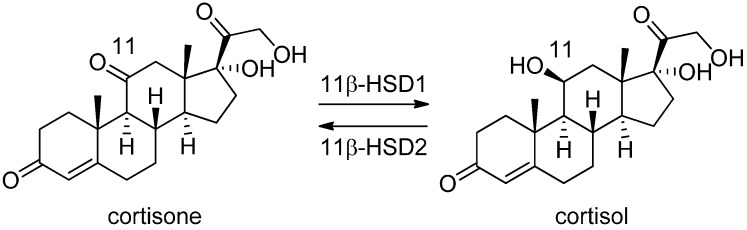
Interconversion of cortisone and cortisol catalyzed by the 11β-HSD enzymes.

The two isoenzymes of 11β-HSD catalyze the interconversion of the biologically inactive cortisone and the active cortisol (Figure 6). The 11β-HSD1 is ubiquitously expressed and mediates the regeneration of active glucocorticoids [77,78], whereas 11β-HSD2 catalyzes the inactivation of glucocorticoids mainly in the kidney, colon and placenta. There is evidence for beneficial effects of 11β-HSD1 inhibition in the metabolic syndrome [79,80,81,82,83,84,85,86,87], atherosclerosis [88,89,90,91], osteoporosis [66,92], glaucoma [93,94,95], cognitive functions [96,97,98,99,100], skin aging [101], and wound healing [102,103]. Thus, inhibition of 11β-HSD1 has substantial therapeutic potential for glucocorticoid-related diseases. Numerous 11β-HSD1 inhibitors have already been identified and some have reached the clinical phase, but to date still no 11β-HSD1 inhibitor is on the market [104]. Although structural variety is prevalent among the 11β-HSD1 inhibitors, the crystal structures are rather similar [105]. Nevertheless, the observed differences are useful in selecting a structure for further *in silico* evaluations. To date, 27 human, four mouse, and three guinea pig 11β-HSD1 crystal structures are accessible through the PDB; however, there is currently no 3D structure of human 11β-HSD1 in -complex with a substrate available. In addition, structural information about 11β-HSD2 is entirely missing.

**Figure 7:**
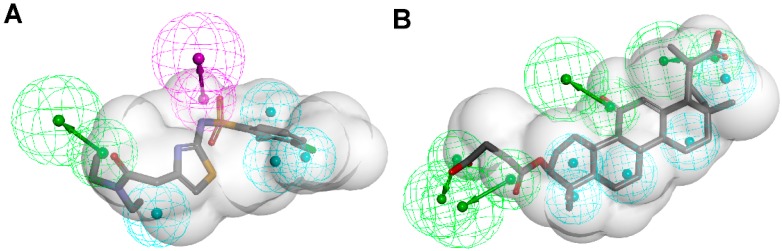
The selective (**A**) and nonselective (**B**) 11β-HSD1 pharmacophore models reported in the study by Schuster and Maurer [106]. The training compounds CAS 376638-65-2 (**A**) and carbenoxolone (**B**) are aligned to the models. The 11β-HSD1-selective model consisted of four H features (blue), one HBA (green) and one HBD (magenta) feature and a shape restriction. The nonselective 11β-HSD model contained five H, four HBA features and also a shape restriction.

Schuster and Maurer *et al.* [106] were the first to introduce pharmacophore models for the identification of novel classes of 11β-HSD1 inhibitors. As there was no X-ray crystal structure of 11β-HSD1 available at the beginning of their study, they employed two ligand-based pharmacophore models as VS tools. Depending on the 11β-HSD activity of the training compounds used for the model generation, a model for 11β-HSD1-selective (Figure 7A) and one for nonselective 11β-HSD inhibitors (Figure 7B), preferably targeting 11β-HSD2, were developed. These models identified compounds resembling the structure of the known unselective 11β-HSD inhibitor glycyrrhetinic acid (GA), steroid-like compounds, and novel structural classes. A comparison of the training set compounds used for the generation of the 11β-HSD1-selective and the 11β-HSD-nonselective pharmacophore models with the compounds from the VS showed similar inhibition profiles towards 11β-HSD1 and 11β-HSD2.

Testing the inhibitory potential of their VS hits, Schuster and Maurer *et al.* determined biological activities for human 11β-HSD1, 11β-HSD2, 17β-HSD1 and 17β-HSD2 [106]. Out of 30 tested compounds, seven inhibited 11β-HSD1 activity by more than 70% at 10 µM and only three showed reasonable selectivity over the other tested enzymes.

The potential of the selective 11β-HSD1 ligand-based pharmacophore model obtained by Schuster and Maurer *et al.* [106] was further evaluated by Hofer *et al.* [107]. VS and subsequent lead optimization by classical bioisosteric studies revealed a class of selective 11β-HSD1 inhibitors bearing an arylsulfonylpiperazine scaffold. Docking studies, performed to rationalize the biological data, showed good alignment of all active compounds with the co-crystallized ligand, belonging to the same chemical scaffold. This structure-based approach further validated the ligand-based pharmacophore model.

**Figure 8:**
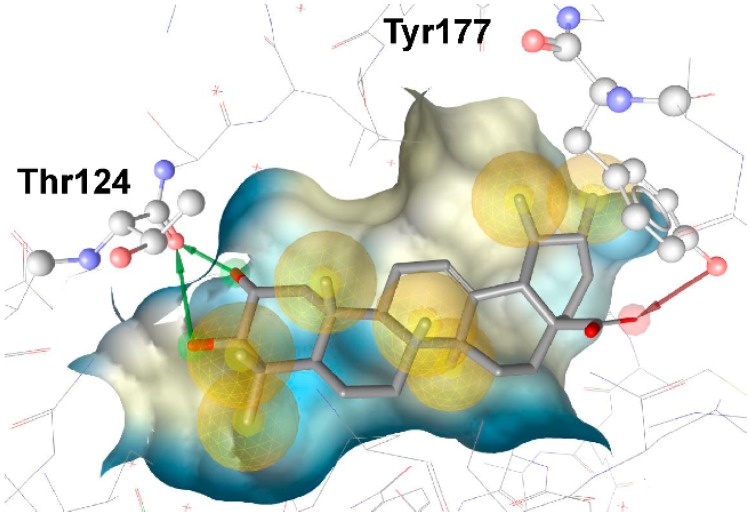
The docking pose of the potent inhibitor corosolic acid in the binding pocket of 11β-HSD1 (PDB entry 2BEL [110]) suggests interactions with Thr124 and Tyr177.

Rollinger *et al.* used the same pharmacophore model as a query for the screening of a database consisting of constituents from medicinal plants, in order to identify natural compounds selectively inhibiting 11β-HSD1 [108]. The chemical class of triterpenoids displayed one of the dominating chemical scaffolds in the virtual hit list. Earlier investigations led to the assumption that extracts from the anti-diabetic medical plant loquat (*Eriobotrya japonica*) dose-dependently and preferentially inhibit 11β-HSD1 over 11β-HSD2 [109]. Therefore, the virtual screening hit corosolic acid, a known constituent of *E. japonica*, was tested and identified as potent inhibitor of human 11β-HSD1 with an IC_50_ of 810 nM [108]. Subsequent bioassay-guided phytochemical analyses revealed further secondary metabolites from the triterpenoid ursane type as 11β-HSD1 inhibitors with IC_50_ in the micromolar range. Importantly, a mixture of the constituents with moderate inhibitory activities displayed an additive effect. This is a common observation in phytotherapy, where a mixture of constituents is often responsible for the therapeutic effect. Docking studies for binding mode prediction suggested a flipped binding mode, where these triterpenoids would not interact with the catalytic amino acids but with Thr124 and Tyr177 (Figure 8). Based on the most active compounds, a pharmacophore model was generated that enriched active molecules on the top of the hit list and successfully reflected the substructures important for binding. Additionally, this study demonstrates a further application in the drug discovery process—finding inhibitors from natural origins.

**Figure 9:**
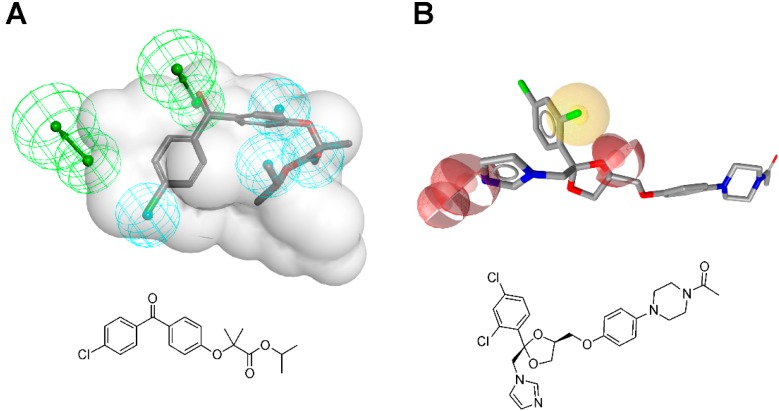
Both the refined 11β-HSD1 (**A**) and 11β-HSD2 (**B**) model identified novel scaffolds [29]. The inhibitor fenofibrate maps the 11β-HSD1 model (**A**) and ketoconazole matches the 11β-HSD2 model (**B**). Both models were screened with one omitted feature. The 2D structures of the novel inhibitors are depicted underneath the alignments.

Considering the ongoing search for novel 11β-HSD1 inhibitors, high predictivity and performance of pharmacophores are essential. Thus, to maintain high quality standards, pharmacophore models have to be continuously re-evaluated and improved. Vuorinen *et al.* [29] performed a refinement study of the 11β-HSD pharmacophore models previously described by Schuster and Maurer *et al.* [106] and Kratschmar *et al.* [78]. In a first step, the selective 11β-HSD1 model was refined by exchanging a chemical feature and removing shape restriction using literature data. Whereas the unrefined model was only able to recognize two out of 14 test compounds, the refined model found 13. Subsequent prospective VS and biological testing revealed better performance of the refined model. However, although the refinement improved the sensitivity of the model and more active compounds were found, it decreased specificity and also more inactive compounds fitted into the model. Adding a shape restriction, following newly identified selective 11β-HSD1 inhibitors, increased specificity, whereas the sensitivity remained the same. For additional testing of the model quality on a different dataset, literature-based validation was performed with structurally diverse compounds, which had not been used in the model development. Specificity was increased, whereas sensitivity decreased. This illustrates that improvement of model quality is accompanied by balancing the specificity and sensitivity of a model. Refinement of the 11β-HSD2-selective model was equally conducted. Since there is no 3D structure of 11β-HSD2 available and only a few selective, mainly triterpenoid scaffold-based 11β-HSD2 inhibitors are known, the 11β-HSD2 model data are biased. They were, however, able to improve 11β-HSD2 model quality, and novel active scaffolds selectively inhibiting both 11β-HSD1 (Figure 9A) and 11β-HSD2 (Figure 9B) were discovered [29].

Using the refined 11β-HSD1 model, Vuorinen *et al.* applied a VS to filter a database consisting of constituents from medicinal plants to identify potential 11β-HSD1 inhibitors focusing on triterpenoids present in *Pistacia lentiscus (P. lentiscus)*, so-called mastic gum that is used in traditional Greek medicine for the treatment of diabetes [111]. The VS hit list contained eight hits of *P. lentiscus* constituents. The two main constituents of mastic gum, masticadienonic acid and isomasticadienonic acid, were chosen for further biological evaluation. Both compounds were shown to selectively inhibit 11β-HSD1 over 11β-HSD2 with IC_50_ values of 2.51 μM for masticadienonic acid and 1.94 μM for isomasticadienonic acid, respectively. Examination of the whole resin’s activity revealed half the IC_50_ value of the single molecules, suggesting an additive inhibitory effect. Thus, the hypothesis of 11β-HSD1 involvement in the antidiabetic activity of mastic gum was supported. Analyzing the binding orientation of the two substances by docking revealed interactions comparable to that of the co-crystallized ligand carbenoxolone, suggesting a competitive binding mode. Thus, the refined pharmacophore model has proven its ability to identify novel 11β-HSD1 inhibitors from natural sources.

Yang *et al.* performed a study using different 11β-HSD1 crystal structures in order to identify synthetic 11β-HSD1 inhibitors [112]. They applied a combined approach of molecular docking and ligand-based pharmacophore modeling. For virtual docking calculations the crystal structure 1XU9 [113] and the program DOCK4.0 [114] were used to screen a commercial compound database. The 3000 compounds with the highest docking score were selected for a second docking run using Glide [115]. Additionally, a ligand-based pharmacophore model for selective 11β-HSD1 inhibitors was constructed using Catalyst 4.10 [116], which was used for screening the 3000 compounds with the Best Flexible Search mode. Compounds with high docking and good fit score were further evaluated by filtering for drug likeness and finally selected for biological testing on human and mouse 11β-HSD1. Importantly, other studies showed significant species-specific variability in the potency of various 11β-HSD1 inhibitors, indicating significant differences in the 3D organization of the hydrophobic substrate-binding pocket of human and mouse 11β-HSD1 [117,118]. Due to this issue, the tested compounds showed different inhibition profiles for the mouse and human enzyme. Eleven out of 121 tested compounds inhibited the human 11β-HSD1 with IC_50_ values of 0.26–14.6 μM, whereas six molecules inhibited the mouse 11β-HSD1 with IC_50_ values of 0.48–12.49 μM. Two substances displayed overlapping hits with IC_50_ for the human 11β-HSD1 of 0.69 μM and 3.57 μM and for the mouse isoenzyme of 0.48 μM and 2.09 μM, respectively. In order to test the selectivity over 11β-HSD2 for subsequent animal studies, only compounds inhibiting mouse 11β-HSD1 were tested for the inhibition of mouse 11β-HSD2. All compounds selectively inhibited 11β-HSD1. Nevertheless, selectivity assessment needs to include human 11β-HSD2 and, ideally, other SDRs. Cross-species activity would be the optimal situation for preclinical evaluation in the development of novel drug candidates.

**Figure 10:**
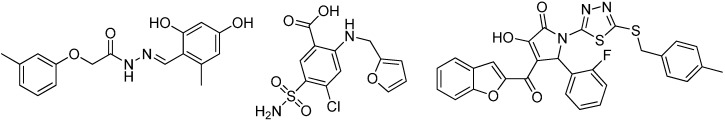
The three new identified scaffolds by Yang *et al.* [119].

A consecutive *in silico* study of Yang *et al.* includes virtual screening with 11β-HSD1 structure-based pharmacophore models and subsequent docking for hit selection [119]. Compounds chosen in the docking process were able to form hydrogen bonds with the amino acids Tyr183 and Ser170 from the catalytic triade. Nine out of 56 enzymatically tested compounds exhibited dose-dependent and selective inhibition of human 11β-HSD1 with IC_50_ values between 0.85–7.98 µM and six substances inhibited the mouse 11β-HSD1 with IC_50_ values between 0.44 µM and 8.48 µM. Four substances inhibited both isoenzymes with similar IC_50_ values. In contrast, during their first 11β-HSD1 *in silico* study, Yang *et al.* identified 11 out of 121 tested compounds from the same database as actives against 11β-HSD1, with IC_50_ values between 0.26–14.6 µM [113]. Four of the identified 11β-HSD1 inhibitors incorporate an arylsulfamido scaffold, an already reported scaffold to inhibit 11β-HSD1 [118]. Besides, three new scaffolds were identified as displayed in Figure 10.

Table 1 summarizes the pharmacophore-based virtual screening studies and illustrates the scaffold-hopping of the different 11β-HSD1 inhibitors.

#### 3.1.1. 17β-Hydroxysteroid Dehydrogenase Type 1

**Figure 11:**
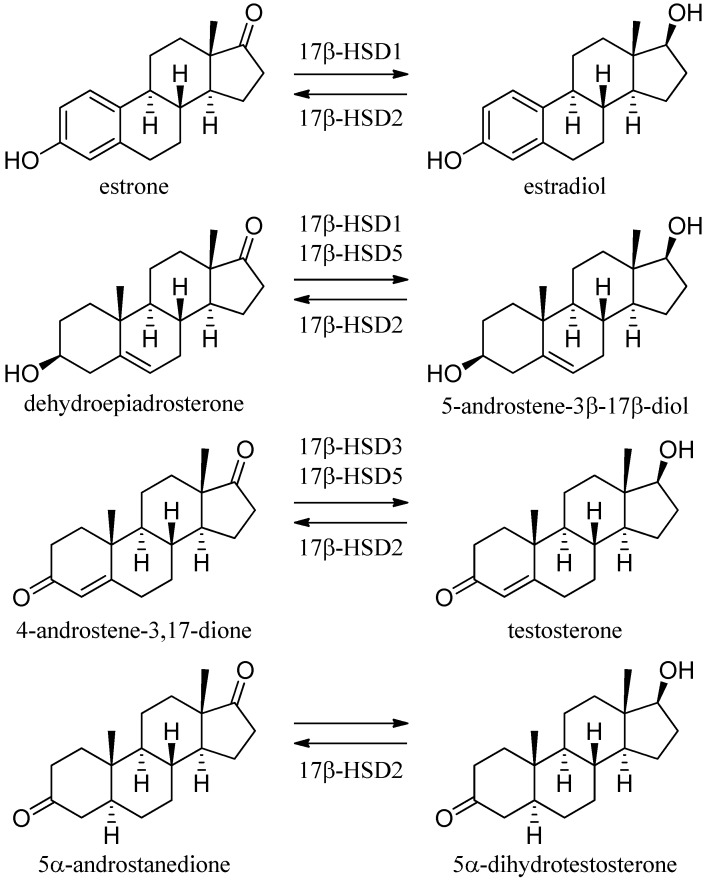
17β-HSDs involved in sex steroid metabolism.

To date, 14 different human 17β-hydroxysteroid dehydrogenase (17β-HSD) enzymes have been reported, all of which except the aldo-keto reductase (AKR) member 17β-HSD5 (AKR1C3) belong to the SDR family [120]. The 17β-HSDs essentially regulate the local metabolism and activity of estrogens and androgens (Figure 11).

**Table 1:** 11β-HSD1 pharmacophore-based virtual screening studies summarized.

Reference Study Aim	Pharmacophore Model	Database Used for VS	Hits				Biological Testing		
			Most Active Hit	Number of Virtual Hits	Tested *in Vitro*	Actives	Assay	IC_50_	Selectivity
Schuster and Maurer *et al.* [106] *11β-HSD1 inhibitors*	Ligand-based using Catalyst	Asinex Gold and Platinum, Bionet 2003, ChemBridge DBS, Clab and IDC, Enamine 03, Interbioscreen 03 nat and syn, Maybridge 2003, NCI, Specs 09 03	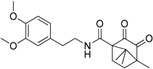	16/20304	15	2	Lysate	2.03 and 7.59 μM	Against 11β-HSD2, 17β-HSD1, and 17β-HSD2
	11β-HSD1 selective (4 H, 1 HBA, 1 HBD, and shape restriction)								
	11β-HSD unselective (5 H, 4 HBA and shape restriction)		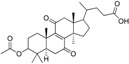	107/1776579	15	5	Lysate	11β-HSD1 0.144–2.81 μM 11β-HSD2 0.06–3.95 μM	Most of them against 17β-HSD1 and 17β-HSD2
Hofer *et al.* [107] *Lead optimization*	11β-HSD1 selective from Schuster and Maurer *et al.* [106]	In-house database	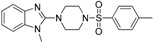	-	-	-	Lysate	0.7 μM	Against 11β-HSD2
Rollinger *et al.* [108] Natural compounds inhibiting *11β-HSD1*	11β-HSD1 selective from Schuster and Maurer *et al.* [106]	DIOS (Natural products in-house database)	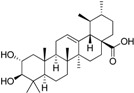 corosolic acid	172	1	1	Lysate	0.81 μM	Against 11β-HSD2
Vuorinen *et al.* [29] *Refinement study*	Refined models from Schuster and Maurer *et al.* [106] Using Discovery Studio	In-house database, DIOS	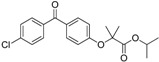 fenofibrate	463	9	3	Lysate	Considered as active if remaining enzyme activity ≤55% at test substance concentration of 20 μM or ≤65% at test substance concentration of 10 μM 5%–40%	Two preferentially inhibited 11β-HSD2, one was unselective
	11β-HSD1 selective								
	11β-HSD2 selective	In-house database, Specs, Maybridge	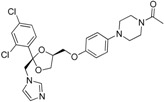 ketoconazole	444	25	2		11%–61% Enzyme rest activity	One preferentially inhibited 11β-HSD1 and one was unselective
	11β-HSD unselective	EDC, In-house database		38	4			36%–49% Enzyme rest activity	Two preferentially inhibited 11β-HSD1 one preferentially inhibited 11β-HSD2
Vuorinen *et al.* [112] *Mode of action study*	Refined 11β-HSD1 model from Vuorinen *et al.* [29]	DIOS	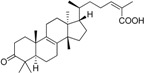	305/6702	2	2	Lysate	1.94 μM and 2.15 μM	Against 11β-HSD2
Yang *et al.* [113] *11β-HSD1 inhibitors*	Ligand-based Using Catalyst (4 H, 1 HBA, 1 AR)	SPECS	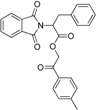 Active against human 11β-HSD1 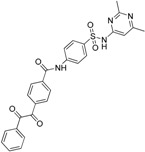 Active against human and mouse 11β-HSD1	3000 Selected by docking (these 3000 were fitted in the pharmacophore model)	121 (39 out of docking and 82 from pharmacophore modeling)	11	Scintillation proximity assay	Human 11β-HSD1 0.26–14.6 μM Nine compounds Mouse 11β-HSD1 0.48–12.49 μM	Only tested against mouse 11β-HSD2 not tested toward the human 11β-HSD2
Yang *et al.* [119] *11β-HSD1 inhibitors*	Two structure-based models using LigandScout (PDB code 2IRW) (3 H, 1HBD, 1 HBA)	SPECS	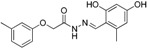	1000 Selected for each model	56	Nine human and six mouse	Scintillation proximity assay	Human 11β-HSD1 0.85–7.98 μM Mouse 11β-HSD1 0.44–8.48 μM	Against 11β-HSD2

The enzyme 17β-HSD1 catalyzes the NADP (H)-dependent reduction of the weak estrogen estrone to the potent estradiol and to a minor extent of dehydroepiandrosterone (DHEA) to 5-androstene-3β,17β-diol [121]. 17β-HSD1 is predominantly expressed in the human placenta, ovaries, and mammary gland, and is of major importance for the peripheral and gonadal estradiol synthesis [122]. Several studies provide evidence for the association of 17β-HSD1 with breast cancer [123,124,125], endometriosis [52,126], endometrial cancer [127] and uterine leiomyoma [128].

**Figure 12:**
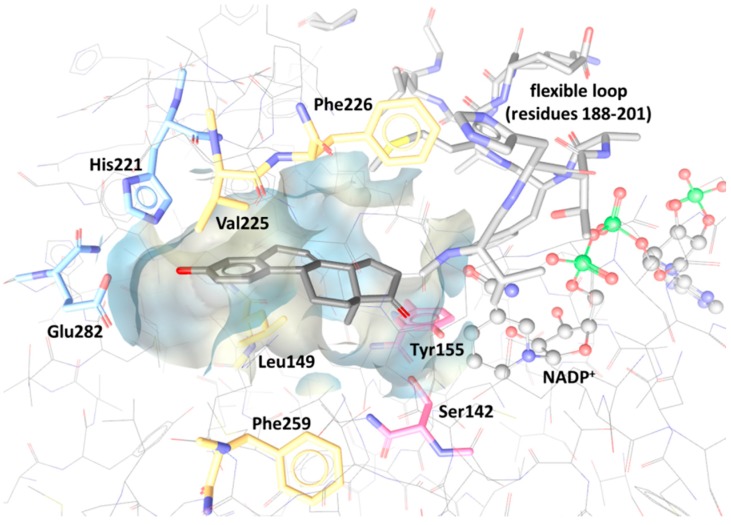
Shape binding site of 17β-HSD1 with equilin as co-crystallized ligand, key residues, a flexible loop and the cofactor NADP^+^ (PDB 1EQU).

Despite the recently increasing numbers of reported 17β-HSD1 inhibitors, still no compound reached clinical trials. To date, more than 20 crystal structures have been published. The binding pocket of 17β-HSD1 is an elongated hydrophobic tunnel, with key roles for Leu149, Val225, Phe226, and Phe259, and polar areas at each end formed by His221 and Glu282 on one side and the catalytically essential residues Ser142 and Tyr155 on the other side. The active site is limited by a flexible loop (amino acids 188–201), which is not well resolved in the crystal structures (Figure 12) [13].

In 2001, Hoffren *et al.* were the first to report structure-based pharmacophore models for the discovery of 17β-HSD1 inhibitors [129]. The pharmacophore models were validated to specifically recognize compounds possessing the structural and chemical features of steroids and flavonoids. Coumestrol displayed the most potent 17β-HSD1 inhibiting activity among the test compounds used for model validation. However, coumestrol also inhibited 17β-HSD5 and is, therefore, not selective [130]. Unfortunately, the virtual hits were not confirmed by biological validation [129].

To support the development of therapeutic inhibitors, database creation for pharmacophore model validation should focus on selective inhibitors to increase model selectivity and sensitivity. Since steroidal inhibitors and natural phytoestrogens, including flavonoids, often exhibit cross-reactivity with other enzymes and hormone receptors involved in the steroidogenesis, non-steroidal scaffolds are more favorable for virtual screening and drug development. However, although highly selective inhibitors are needed for many therapeutic applications, polyvalent inhibitors acting on synergistic pathways may be advantageous in some situations.

**Figure 13:**
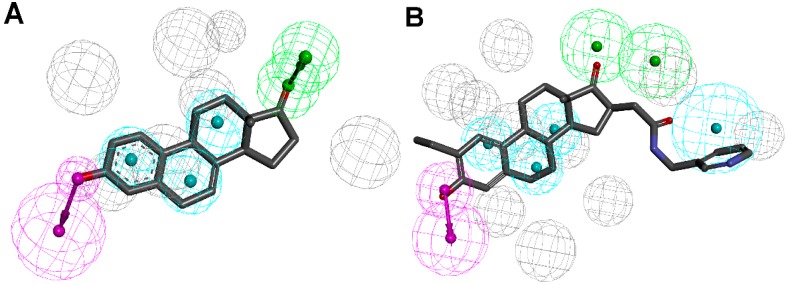
(**A**) 17β-HSD1 model based on the equilin crystal structure (PDB entry 1EQU [5]); (**B**) The potent inhibitor STX 1040 maps the hybrid 17β-HSD1 pharmacophore model [133].

The 17β-HSD1 can be inhibited by several modes: competing reversibly and irreversibly with the natural substrate for its binding site, competing with NADP(H) for its binding site at the Rossmann fold or occupying the ligand and the cofactor binding site by so-called hybrid compounds consisting of a steroidal core and extended side-chains of NADP(H) moieties [131,132]. Since only crystal structures containing steroidal inhibitors were available at that time, Schuster and Nashev *et al.* generated structure-based pharmacophore models based on steroidal inhibitors [133]. They developed two pharmacophore models, representing, on one hand, reversible competitive inhibitors based on the steroidal core equilin (Figure 13A) and, on the other hand, hybrid inhibitors (Figure 13B). Whereas the first model was suggested to be suitable as a general screening tool, expecting many false positive hits, the hybrid model was more restrictive due to the unique scaffold of the underlying hybrid inhibitors. VS and subsequent *in vitro* validation of 14 selected compounds from the virtual hit list revealed, amongst others, two nonsteroidal hits with IC_50_ of 5.7 µM and 19 µM, respectively. As mentioned above, the SDR enzymes share substantial structural similarity. For selectivity assessment, 11β-HSD1, 11β-HSD2, 17β-HSD2, 17β-HSD3 and the AKR 17β-HSD5 were tested. Two additional inhibitors were selective. One was a steroidal compound with an IC_50_ of 3.8 µM for 11β-HSD1 and 47 µM for 17β-HSD1, and one a nonsteroidal 11β-HSD1 inhibitor with IC_50_ of 6.2 µM and comparable activity on 17β-HSD3. These observations emphasize the importance of including structurally related enzymes for selectivity assessment. In addition to the biological selectivity assessment, Schuster and Nashev *et al.* applied pharmacophore models of structurally related enzymes as an alternative strategy to identify unspecific inhibitors [133]. These pharmacophores should act as initial filters to eliminate compounds with a low degree of selectivity that may exhibit off-target effects. Screening the compounds identified as actives for 11β-HSD1 with their previously established selective 11β-HSD1 pharmacophore model resulted in retrieving one hit [106]. By deleting the shape restriction, the second hit was found as well and, at the same time, showed higher best fit values than an inactive compound. Thus, screening of pharmacophore models of related enzymes may facilitate the discrimination of selective and nonselective inhibitors and the virtual hit selection for *in vitro* testing, similar to the study by Guasch *et al.* described above [56].

**Figure 14:**
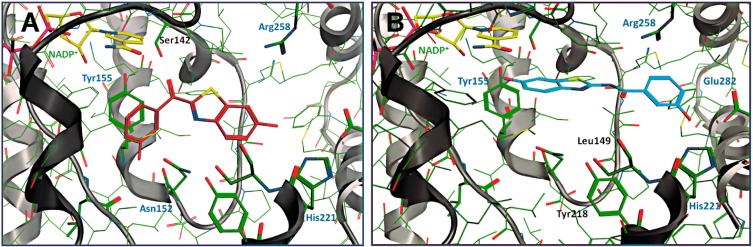
17β-HSD1 in complex with the two hits from Sparado *et al.* [134], (doi:10.1371/journal.pone.0029252.g010, doi:10.1371/journal.pone.0029252.g011) showing a 180° flipped orientation. IC_50_ values of 44 nM (**A**) and 243 nM (**B**).

For pharmacophore model generation, Sparado *et al.* [134] superimposed five 17β-HSD1 crystal structures, covering most of the chemical space occupied by the co-crystallized ligands. Performing a VS of an in-house compound library led to the identification of one virtual hit with moderate inhibitory activity against 17β-HSD1. Application of the rigidification strategy, scaffold hopping and further SAR analysis resulted in two far more potent benzothiazole-scaffold-bearing inhibitors with IC_50_ in cell lysates of 44 and 243 nM, respectively. Both hits were selective against 17β-HSD2. Furthermore, the less active compound still potently inhibited estrogen formation, with a comparable IC_50_ value to the lysates, in a human cell model endogenously expressing 17β-HSD1. The more potent compound showed pronounced affinity to bind to ERα and ERβ. Depending on whether binding to ERα and ERβ results in agonistic or antagonistic effects, this could cause beneficial or adverse effects. Interestingly, although the two hits differ only in a carbonyl and amide bridge, respectively, binding mode investigations by docking showed a 180° flipped orientation of the two molecules (Figure 14). The observation of a flipped binding mode was also discovered for corosolic acid and other triterpenoides in the binding pocket of 11β-HSD1 as described earlier [108]. A follow-up lead optimization study to improve activity and selectivity of the two compounds for *in vivo* applications, without the help of molecular modeling techniques, led to the discovery of two new lead compounds [135]. They showed selectivity over 17β-HSD2, no ER binding and promising activity in the intact cell model.

Table 2 shows a summary of the prospective pharmacophore-based virtual screening studies and illustrates the scaffold-hopping potential for 17β-HSD1 inhibitors.

Structure-based and ligand-based pharmacophore modeling was performed by Karkola *et al.* [136]. They generated four pharmacophore models with different methods based on a crystal structure, a relaxed crystal structure, alignment of thienopyrimidinone inhibitors, and a docked complex of 17β-HSD1 with a potent inhibitor. By VS, they found several compounds fitting into the active site of 17β-HSD1 without determining the activity of the hits. However, to validate these hits as 17β-HSD1 inhibitors, biological testing is needed. In addition, they could apply their differently generated pharmacophore models to calculate selectivity and sensitivity.

#### 3.1.2. 17β-Hydroxysteroid Dehydrogenase Type 2

The oxidative inactivation of estradiol to estrone is predominantly catalyzed by 17β-HSD2. Additionally, 17β-HSD2 is capable of converting testosterone into 4-androstene-3,17-dione (androstenedione), 5α-dihydrotestosterone (DHT) into 5α-androstanedione, 5-androstene-3β,17β-diol to DHEA, and 20α-dihydroprogesterone into progesterone using the cofactor NAD+ [137,138]. The 17β-HSD2 is expressed in various tissues such as bone, placenta, endometrium, breast, uterus, prostate, stomach, small intestine, and colon epithelium [139,140]. The current treatment options for osteoporosis bear several limitations. Since 17β-HSD2 is expressed in osteoblasts, its inhibition may provide a new approach to treat osteoporosis by increasing the local availability of estradiol.

**Figure 15:**
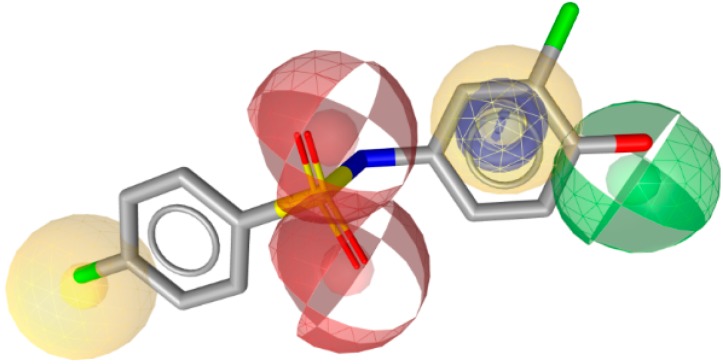
The selective 17β-HSD2 model contains a HBD feature (green sphere), which is important for 17β-HSD2 inhibitors such as the newly identified phenylbenzene-sulfonamide derivative 13 [141].

Since 17β-HSD2 contains an N-terminal transmembrane anchor, the experimental 3D structure determination remains a challenge and, to date, still no crystal structure is available. Due to this lack, Vuorinen *et al.* constructed three ligand-based pharmacophore models as virtual screening filters [141]. Virtual hit-testing in a cell-free assay revealed seven out of 29 compounds with IC_50_ values against 17β-HSD2 ranging between 0.24 µM and 33 µM. Most of the active compounds represented phenylbenzene-sulfonamides and -sulfonates. With the new structural classes of 17β-HSD2 inhibitors, they performed a SAR study using two different approaches: first, by a 2D similarity search without fitting the compounds into the pharmacophore models, and second, using a pharmacophore model for VS. From the 2D search, one out of 16 compounds inhibited 17β-HSD2 with an IC_50_ of 3.3 µM, whereas the VS showed five out of 14 compounds with IC_50_ between 1–15 µM. Selectivity of all active compounds was tested against inhibition of 17β-HSD1, 17β-HSD3, 11β-HSD1, and 11β-HSD2. The activity data of the phenylbenzene-sulfonamide and -sulfonate inhibitors revealed a phenolic hydroxyl group with hydrogen bond donor functionality, which was important for 17β-HSD2 inhibition. This feature was confirmed by a ligand-based pharmacophore model that was developed based on several of the newly identified active compounds (Figure 15). Furthermore, to improve the initial pharmacophore model, a refinement database was created, including the original test set compounds and the newly identified inhibitors as well as the inactive compounds. The specificity of the model was increased by adding exclusion volumes. This approach is an important step to enhance a model’s ability to enrich active compounds from a database.

#### 3.1.3. 17β-Hydroxysteroid Dehydrogenase Type 3

The 17β-HSD3 is almost exclusively expressed in the testes and catalyzes the reduction of androstenedione to testosterone in the presence of NADPH [142]. Although 17β-HSD3 is mainly found in the testes, there is evidence for 17β-HSD3 mRNA up-regulation in prostate cancer [143]. Co-expression of 17β-HSD5, catalyzing the same reaction, might limit the therapeutic efficacy of 17β-HSD3 inhibitors and a combined treatment with inhibitors against both enzymes should be envisaged.

The enzyme is anchored through an N-terminal transmembrane domain to the endoplasmic reticulum, and, like 17β-HSD2, its catalytic domain faces the cytoplasmic compartment [144,145]. As for 17β-HSD2, there is still no crystal structure available for the membrane protein 17β-HSD3.

**Figure 16:**
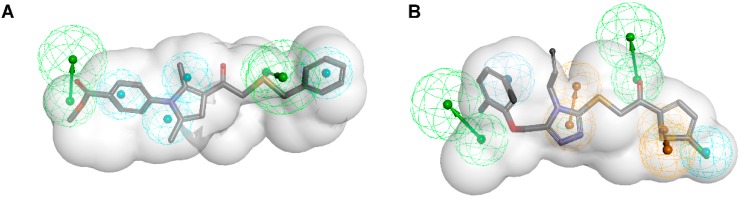
(**A**) The novel 17β-HSD3 inhibitor 1–7 was identified with the steroid-based model consisting of two HBAs (green) and four H features (blue); (**B**) The non-selective inhibitor 2-2 mapped the nonsteroid-based 17β-HSD3 model containing two HBAs, two AR (orange), one H and one H-AR feature [146].

Two ligand-based pharmacophore models, based on steroidal and nonsteroidal 17β-HSD3 inhibitors, were developed by Schuster *et al.* [146] (Figure 16). These ligand-based models supported the observations by Vicker *et al.* of a highly hydrophobic active site of 17β-HSD3 [147]. The models were then used to screen eight commercial databases and the hit list was further filtered prior to the selection of hits. Enzymatic tests showed that, from the steroid-based model, two out of 15 tested substances inhibited 17β-HSD3, with one also inhibiting 17β-HSD1 [146]. At the same time, three other compounds inhibiting the AKR 17β-HSD5 were identified. The 17β-HSD5 is a multifunctional enzyme and, like 17β-HSD3, catalyzes the conversion of androstenedione into testosterone. The most potent compound was not selective and also inhibited 11β-HSD1 and 11β-HSD2. Similar results were obtained with the nonsteroidal model. The nonsteroidal model and its training compounds displayed several overlapping features with the lead compound identified earlier by Vicker *et al.* [147]; thus, the examination of their compounds for 17β-HSD5 inhibitory activity would be interesting. These observations again emphasize the importance of including structurally related enzymes, independently of their enzymatic classes, for selectivity profiling. A summary of the 17β-HSD3 pharmacophore-based virtual screening study presented by Schuster *et al.* is provided in Table 3.

**Table 2:** 17β-HSD1 pharmacophore-based virtual screening studies summarized.

Reference Study Aim	Pharmacophore Model	Database Used for VS	Hits				Biological Testing		
			Most Active Hit	Number of Virtual Hits	Tested *in Vitro*	Actives	Assay	IC_50_	Selectivity
Schuster and Nashev *et al.* [133] *17β-HSD1 inhibitors*	Structure-based Using LigandScout and Catalyst 1I5R model (4 H, 2HBA, 2 HBD) Based on a hybrid inhibitor	NCI, SPECS	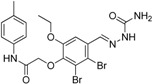	1559/340042	14	4, IC_50_ < 50 μM	Lysates	5.7–47 μM	Selective over 17β-HSD2, 17β-HSD3, 17β-HSD5 and 11β-HSD1, except one compound, which was not selective towards 17β-HSD5 and 11β-HSD1 However, one compound inhibited 17β-HSD3 and 11β-HSD1 but not 17β-HSD1 and another compound inhibited 11β-HSD1 only
Sparado *et al.* [134] *17β-HSD1 inhibitors and lead optimization*	Ligand-based By superimposing co-crystallized ligands using MOE (5 H, 3 HBA, 1 HBD, 1 AR)	In-house database	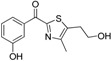	-/37	-	1	Cell-free	34% Enzyme inhibition with 10 μM test compounds	Selectivity of optimized compounds tested against 17β-HSD2 and ERα and ERβ

**Table 3:** Summary of the 17β-HSD3 pharmacophore-based virtual screening study.

Reference Study Aim	Pharmacophore Model	Database Used for VS	Hits				Biological Testing		
			Most Active Hit	Number of Hits after Filtering	Tested *in Vitro*	Actives	Assay	Enzyme Inhibition	Selectivity
Schuster *et al.* [146] *17β-HSD3 inhibitors*	Ligand-based Using Catalyst	Asinex Gold and Platinum, ChemBridge, Enamine, IF-Labs, Maybridge, Specs, Vitas-M	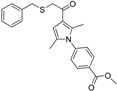	3921/1712102	15	2	Lysates	Inhibition >40% with 2 μM test compounds as threshold 41.3% and 50.8%	Selective over 17β-HSD2, 17β-HSD4, 17β-HSD7, 11β-HSD1, and 11β-HSD2, acceptable selectivity over 17β-HSD1 and 17β-HSD5. However, several hits inhibited 17β-HSD5 more potently than 17β-HSD3
	Model 1: steroidal training compounds (four H, two HBA)								
	Model 2: non-steroidal training compounds (one H, two HBA, two AR, one H-AR)		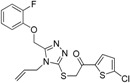	8190/1712102	16	2		55.6% and 57.5%	Selective over 17β-HSD2, 17β-HSD4, 17β-HSD7, and 11β-HSD2, acceptable selectivity over 17β-HSD1 One hit was not selective over 17β-HSD5 and the other not over 11β-HSD1. However, several hits inhibited 17β-HSD5 more potently than 17β-HSD3

### 3.2. Applications in Toxicology

#### 3.2.1. Anti-Target Screening

Although the actual virtual screening process is analogous to lead identification, anti-target screening pursues a different aim. Lead identification focuses on the discovery of ligands for therapeutically relevant targets, whereas anti-target screening aims at predicting the interaction of molecules with macromolecules mediating potentially harmful effects (so-called anti-targets). These investigations support the identification of (serious) adverse events already at an early stage in drug development. This strategy is powerful, as recently shown by Kratz *et al.* [148], who successfully applied pharmacophore models to identify inhibitors of the human ether-a-go-go-related gene (hERG) potassium channel, thereby predicting the cardiotoxic potential of the investigated molecules [148].

#### 3.2.2. Parallel Screening

Parallel screening represents an extension to lead identification and anti-target screening protocols. It investigates not a single target but a whole collection of macromolecules with the aim of obtaining activity profiles of compounds of interest in order to prioritize further investigation. Thus, the focus of this technique shifts from the target of interest to the compound of interest, which is screened against a collection of pharmacophores, representing a plethora of different targets. Parallel screening has the potential to identify macromolecular interaction partners of the investigated molecule, thereby providing novel insight into its biological activities. These activities may include beneficial (*i.e.*, therapeutic) and harmful (*i.e.*, toxic) effects. Therefore, the results support the evaluation of a compound both with regard to the occurrence of adverse events and potential novel application fields (whenever this aspect represents the main aim of the parallel screening, this technique is also referred to as drug repurposing or drug repositioning). In the attempt to explore the biological activity of leoligin, a lignan isolated from the alpine plant Edelweiss (*Leontopodium alpinum*), the compound was screened against the Inte:Ligand pharmacophore collection in the course of a parallel screening [149]. Among the proposed targets, wascholesteryl ester transfer protein (CETP), a target involved in lipoprotein metabolism, was shown to be activated by leoligin in subsequent experimental testing. On the other side, leoligin was also predicted to inhibit the cytochrome P450 (CYP) isoforms 1A2, 2C9, and 3A4 [150], which are involved in the metabolic clearance of exogenous compounds. While it was not active on CYP1A2, it was a weak inhibitor on CYP2C9, and a sub-micromolar IC_50_ was determined for CYP3A4 [150]. Inhibition of CYP enzymes can cause severe drug-drug interactions that may lead to serious adverse effects and eventually require the termination of a drug development project. Accordingly, both potentially beneficial (CETP activation) and potentially harmful (CYP inhibition) effects can be detected during a parallel screening.

#### 3.2.3. Examples

There is a great demand for improved methods for the safety assessment of man-made chemicals released into the environment [68,151]. Endocrine-disrupting chemicals (EDCs) are exogenous substances interfering with hormone synthesis, metabolism and/or hormonal regulation, thereby adversely affecting human health by contributing to developmental and reproductive disorders, cardio-metabolic diseases, cancer, and immune-related diseases and psychiatric disorders [152]. EDCs include substances used in agriculture, industrial production, dyes, food preservatives, or body care products and cosmetics. Several SDRs are essentially involved in the control of the local availability of active glucocorticoids, androgens and estrogens, and these enzymes should therefore be considered in the assessment of potential EDCs. *In silico* tools are well established in the drug discovery process; however, they can also display a valuable part in the identification of new EDCs or the mechanism of action of known EDCs [68].

**Figure 17:**
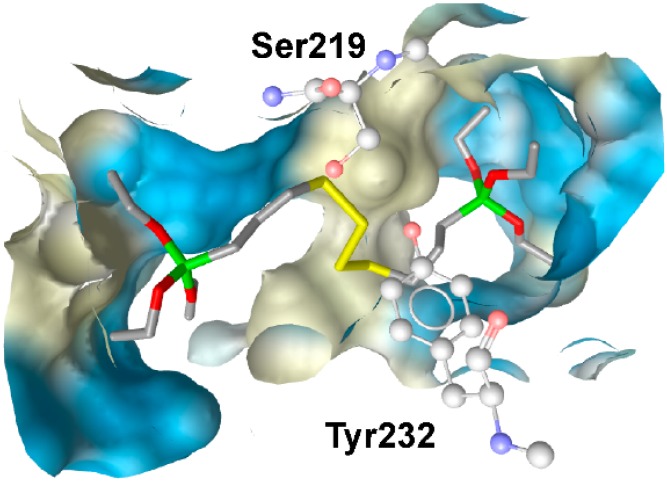
Docking of silane into the homology model of 11β-HSD2 [78] suggests hydrogen bond interactions with Ser219 and Tyr232 [70].

Nashev and Vuorinen *et al.* [70] reported a pharmacophore-based virtual screening using a ligand-based 11β-HSD pharmacophore model preferentially focusing on 11β-HSD2 [106]. The 11β-HSD2 protects the mineralocorticoid receptor (MR) from activation by cortisol and renders specificity for the much less abundant aldosterone to activate this receptor. Genetic defects of this enzyme cause the syndrome of apparent mineralocorticoid excess (AME), characterized by hypokalemia, hypernatremia, and severe hypertension [153,154]. In addition, placental 11β-HSD2 protects the fetus from enhanced maternal cortisol exposure [155,156]. Therefore, disrupting corticosteroid action by EDCs can be expected to cause substantial adverse health effects. VS of an EDC database predicted 29 compounds fitting into the model of which five hits were selected for biological evaluation. Two compounds were found to inhibit 11βHSD2, the silane coupling agent AB110873 and the antibiotic lasalocid, with IC_50_ values of 6.1 µM and 14 µM, respectively. The silane AB110873 is widely used as a rubber additive for the production of tires, mechanical goods, or shoe soles and lasalocid is used as a feed additive for the prevention of infections in the breeding of chicken and turkeys. Docking studies were implemented to understand the binding mode of AB110873 in 11β-HSD2 (Figure 17) and MR.

**Figure 18:**
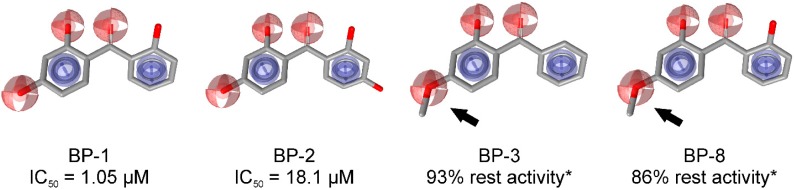
SAR analysis revealed that the etherification of the hydroxyl group (as indicated by the arrows) was responsible for the loss of activity observed for BP-3 and BP-8 [74]. *Remaining enzyme activity at a compound concentration of 20 µM compared to vehicle control.

Genetic defects resulting in 17β-HSD3 deficiency cause 46,XY disorder of sex development [142,157,158]. Inhibition of 17β-HSD3 activity by EDCs might reduce plasma testosterone levels, thereby interfering with male sexual development and contributing to male reproductive disorders. To identify potential EDCs inhibiting 17β-HSD3, Nashev and Schuster *et al.* generated a ligand-based pharmacophore model [74]. VS of an EDC database predicted several organic UV filters containing a benzophenone as a bioactive chemical scaffold. UV filters are a structurally diverse class of chemicals widely used in sunscreens and cosmetics as well as plastic additives. *In vitro* testing of selected virtual hits and similar environmentally relevant derivatives led to the identification of benzophenone-1 (BP-1) as the most potent 17β-HSD3 inhibitor with an IC_50_ of 1.05 µM in intact cells. BP-2,3-benzylidene camphor (3-BC) and 4-methylbenzylidene camphor (4-MBC) moderately inhibited 17β-HSD3 with IC_50_ values between 10.7 µM and 33.3 µM, but showed substantial inhibitory activity on 17β-HSD2 with IC_50_ between 5.9 µM and 10.3 µM. Importantly, the most active compound, BP-1, as well as 3-BC and 4-MBC were not included in the initial virtual hit list but added to the biological testing due to their use as UV filters. Hence, VS displays an initial filter for the identification of potential EDC compound classes and aims at prioritizing the compounds to be included for biological investigations. In analogy to the drug discovery process, it is important to test structurally related enzymes in order to know whether they are affected by a given EDC. Importantly, major metabolites should also be included in the analysis. For example, BP-3 showed no activity against 17β-HSD3, but it is demethylated *in vivo* to the potent inhibitor BP-1 [159]. To explain the differential inhibitory activities of the tested UV filters, Schuster and Nashev conducted pharmacophore-based SAR studies, suggesting that the ether group on BP-3 and BP-8 instead of a hydroxyl group on BP-1 and BP-2 was the reason for the loss of activity of BP-3 and BP-8 (Figure 18). To further study the toxicological relevance of 17β-HSD3 inhibition by BP-1, concentrations reached *in vivo*, especially in the testes, need to be determined.

## 4. Limitations

As with every method, pharmacophore modeling and pharmacophore-based virtual screening also have their limits. A recent study compared the performances of two pharmacophore modeling programs, LigandScout and Discovery Studio, on the identification of novel cyclooxygenase inhibitors [160]. Intriguingly, although both programs succeeded in the identification of novel bioactive molecules, the virtual hit lists retrieved with the two tools were highly complementary. It is of note that not a single overlap in the hit lists was observed, even when the identical crystal structure of a ligand-target complex was employed for model generation. This illustrates that neither of the two programs was capable of comprehensively covering the active space and that models from different programs need to be combined whenever a more complete retrieval of active molecules is required. The authors suggested that the reasons for this finding may be found in the different screening algorithms and feature definitions deployed by the programs.

Feature definitions can be improved in general, as highlighted by the treatment of halogens. Some pharmacophore modeling programs consider halogens solely as hydrophobic moieties in the default settings [7,8,161]. LigandScout, in addition, matches fluorine to HBA features [6]. In 2013, a study by Sirimulla *et al.* [162] revealed that in many ligand-target complexes, halogens participate in strong halogen bond formation, e.g., with aromatic rings, and thereby considerably contribute to the interaction between the ligand and the target. These types of interactions, although often employed by medicinal chemists to improve the binding affinity of compounds [162], are not yet implemented in common pharmacophore-based virtual screening tools.

A major limitation, not only for pharmacophore modeling but for virtual screening tools in general, is the fact that the quality of a pharmacophore model critically depends on the data employed for model generation, refinement, and theoretical validation. Many public data repositories are available that can be explored to build a model. However, caution is required as, apparently, parts of the data are erroneous. Fourches *et al.* investigated six different datasets, and after curation, up to 10% of the original structures were removed [163]. Besides several other preventive measures, the authors suggest to include a final manual inspection step to check the structures of the input compounds. We fully support this recommendation and would even go one step further: Not only the structures of the compounds included in the modeling dataset need to be critically evaluated, but also the annotated biological data. This starts with inclusion/exclusion criteria for appropriate/inappropriate testing systems applied to determine the biological activity of a compound (for example, data obtained from intact cell assays or from animal tissue preparations are of limited use for human enzyme models), the application of suitable activity cut-offs (distinguishing between specific and unspecific effects, depending on the investigated target), and ends at a critical comparison with the original literature as errors can also happen during the transfer of data to depositories. These procedures may be quite elaborate; nevertheless, they are crucial for the generation of high quality models and every modeler is well advised to carefully review the data on which the models are based.

Another limitation is a lack of compounds confirmed as inactive for a specific target. Results on proven inactives for model validation are often not accessible because, unfortunately, negative results are rarely published. The information from confirmed inactive compounds is important for the balancing between selectivity and sensitivity of a model during the validation step. In the drug development process, restrictive models are required because, finally, only one or a few lead compounds are selected for further optimization steps, whereas in a toxicology screening, it is important to correctly find preferably all of the potentially harmful substances. Albeit considerably more successful than random screening, the success rate of VS may still be a limiting factor for toxicological projects. However, it has the ability to identify structural compound classes that then can be further evaluated. Obviously, the database used for VS might be self-limiting as not all potential active compounds are included.

One caveat of pharmacophore modeling is that a modeler needs to be aware of concerns about detailed interaction patterns of the active compounds in the dataset. Although high quality experimental data confirming their binding and activity may be available for these ligands, the exact binding site is still not clearly defined for most of them. Many molecules may occupy a similar yet slightly different part of the binding pocket, e.g., compared to the co-crystallized ligand in an X-ray crystallographic complex employed for model generation. Accordingly, the interaction patterns may differ. This factor is even more pronounced in ligands affecting the function of a protein by binding to allosteric sites, disrupting conformational changes, or interfering with post-translational modifications. Similar concerns also apply for the experimental validation of the *in silico* predictions, as it is, in the end, often not known whether the newly identified compounds indeed exert the predicted binding mode [164]. An X-ray crystal structure of the ligand-target complex would provide the ultimate confirmation of the exact interaction patterns; however, 3D structure resolution of transmembrane proteins by crystallization remains difficult. Several SDRs belong to this class of proteins such as, for instance, 11βHSD2, 17βHSD2, and 17βHSD3. For these proteins, structure-based pharmacophore modeling is currently not possible, and homology modeling remains challenging due to the low sequence similarity of SDRs. However, for these cases, ligand-based pharmacophore modeling displays an elegant solution.

Pharmacophore-based VS proved to be a powerful tool to support drug discovery and development, especially concerning the enrichment of active molecules among test compounds. Nevertheless, expectations concerning the results of VS need to remain realistic. Although sometimes potent compounds are discovered via VS, the majority of virtual hits usually display only weak activity. For this concern, the initial virtual hits from VS should be considered similar to initial experimental hits discovered in a HTS campaign, which also require further chemical optimization steps to develop to potential drug candidates [164].

## 5. Conclusions

The current work summarizes prospective pharmacophore-based studies conducted in the field of steroid biology, with special focus on SDRs, and highlights success stories reported in this area. Pharmacophore models are suitable to address a wide range of issues relevant for both drug discovery and toxicology. This is of special relevance for SDRs, because members of this target class are both associated with therapeutic value (e.g., 17β-HSD1 inhibition for the treatment of hormone-sensitive cancers) and toxicological liabilities (disruption of 11β-HSD2 actions). Although the method itself still has room for improvement as pointed out in the “Limits” section, the caveats associated with pharmacophore modeling largely also apply for other virtual screening techniques. In addition, in case of a lack of available structural data on macromolecular targets, ligand-based modeling strategies offer a useful alternative. The identification of structurally diverse molecules may, to a certain extent, be restricted to the data employed for model generation and refinement. However, the extraction of crucial interactions and their representation via abstract chemical features proved to be a powerful approach to step beyond the initial chemical space. As highlighted in this review, pharmacophore-based VS is a valuable scaffold-hopping tool. Importantly, this allows for the application of pharmacophore-based virtual screening also for compound classes that do not fall into the category of “small drug-like compounds” or whose properties differ from that of synthetic compounds: For example, natural products provide a vast resource for bioactive compounds that can be exploited for therapeutic purposes. On the other hand, the *in silico*-driven investigation of environmental chemicals, which often chemically differ from drug-like molecules, facilitates the rapid identification of potentially harmful compounds that need to be prioritized for experimental evaluation. Given the many application fields of pharmacophore-based virtual screening and the successful examples summarized in this review, an increasing number of studies, also in the field of SDR research, can be expected in the future.

## Abbreviations

The following abbreviations are used in this manuscript:

### Abbreviations

3-BC: 3-benzylidene camphor
4-MBC: 4-methylbenzylidene camphor
AKR: aldo-keto reductase
AME: apparent mineralocorticoid excess
AR: aromatic features
BP: benzophenone
CETP: cholesterylester transfer protein
CYP: cytochrome P450
DHEA: dehydroepiandrosterone
DHT: 5α-dihydrotestosterone
DUD-E: Directory of Useful Decoys, Enhanced
EDCs: endocrine disrupting chemicals
ER: estrogen receptor
GA: glycyrrhetinic acid
H: hydrophobic feature
HBA: hydrogen bond acceptor
HBD: hydrogen bond donor
hERG: human ether-a-go-go related gene
HSD: hydroxysteroid dehydrogenase
HTS: high-throughput screening
IUPAC: International Union of Pure and Applied Chemistry
MD: molecular dynamics
MOE: Molecular Operating Environment
MR: mineralocorticoid receptor
NADP: nicotinamide adenine dinucleotide phosphate
PAINS: Pan-Assay Interference Compounds
PDB: Protein Data Bank
PPAR γ: peroxisome proliferator-activated receptor γ
ROC-AUC: area under the receiver operating characteristics curve
SAR: structure-activity relationship
SDR: short-chain dehydrogenase/reductase
VS: virtual screening
XVols: exclusion volumes
